# Sericin in the isolating solution improves the yield of islets isolated from the pancreas

**DOI:** 10.1007/s10616-016-9970-5

**Published:** 2016-04-28

**Authors:** Shigehiro Yokoi, Makoto Murakami, Mitsuhiro Morikawa, Takanori Goi, Akio Yamaguchi, Satoshi Terada

**Affiliations:** 1 First Department of Surgery, School of Medicine, Faculty of Medical Sciences, University of Fukui, 23-3 Matsuokashimoaizuki, eiheiji-cho, Yoshida-gun, Fukui, 910-1193 Japan; 2 Department of Applied Chemistry and Biotechnology, University of Fukui, 3-9-1 Bunkyou, Fukui, Fukui 910-8507 Japan

**Keywords:** Pancreatic islet isolation, Silk, Sericin, Cytoprotection, Islet transplantation

## Abstract

Approximately half of the transplantable pancreatic islet tissue is lost during isolation, including the digestion and purification steps. Modifying the isolation method could increase the yield. This would enable the one donor-one recipient concept and improve the therapeutic effects of islet transplantation. This study aims to improve islet transplantation by increasing the yield of islets from the pancreas, both the number of islets and their size. Therefore, we used a sericin-containing isolating solution. Rat pancreatic islets were isolated by collagenase digestion and hand picking. We refer to islets isolated with or without sericin in the isolation solution as the sericin and control group, respectively. Volume yield, endocrine function, and islet morphology were compared between the groups. Histological distribution of sericin was evaluated by immunofluorescence staining to examine its mechanism of action in pancreatic islets. The pancreatic islet yield in the sericin group was significantly higher than that in the control group. The endocrine function of islets in the sericin group was comparable to that of islets isolated by conventional methods. Sericin adhered to the surface of isolated pancreatic islets and colocalized with E-cadherin, a cell membrane protein, which might explain the cytoprotective effects of sericin. The islet morphology tended to be better preserved in the sericin group. Sericin could prevent cytoarchitectural damage during the isolation and purification process, resulting in increased pancreatic islet yield. This suggests that sericin could contribute to islet therapy by enhancing the stability of islets.

## Introduction

Islet transplantation is a fundamental treatment method to improve glucose metabolism control in patients with type 1 diabetes mellitus. Najarian et al. (1977) introduced islet transplantation clinically for the first time in 1974. In Japan, pancreatic islet isolation and islet transplantation have been introduced in 2003 and 2004, respectively, and pancreatic islet isolation was performed in 67 cases as of October 2012, and pancreatic islet transplantation was performed in 18 cases. Unlike in western countries, there is a shortage of donors in Japan. In 2000, the Edmonton group proposed a steroid free, rapamycin-based immunosuppressive protocol that uses two or more pancreas donors to supply a sufficient number of islets (Shapiro et al. 2000). However, it is difficult to obtain donors, as most people in Japan are unwilling to participate. Thus, a sufficient amount of isolated pancreatic islets with excellent endocrine function is required for a successful pancreatic islet transplantation and is essential to achieve a one donor-one recipient ratio instead of a culture or cryopreservation. It is known that physical and/or chemical damages by enzymes decrease the yield by 50 % or more during the process of pancreatic islet isolation (Goto et al. 2004; O’Gorman et al. 2005), indicating that the method should be modified.

Silk produced by silkworms consists mainly of the proteins sericin and fibroin. Sericin surrounds fibroin, forming a coating that wraps around the fibroin, thereby strengthening the silk (Voegeli et al. 1993). When silkworm cocoons are used for textiles, sericin is generally removed from the cocoon and discarded. There are numerous reports on the various qualities of sericin; it has anti-oxidant and anti-apoptotic properties, is protective against ultraviolet damage, can accelerate the proliferation of various cells, and provide cytoprotective effects (Sasaki et al. 2000; Sato et al. 2011; Takahashi et al. 2003; Terada et al. 2002; Zhaorigetu et al. 2003, 2007). We previously performed culture preservation and cryopreservation of pancreatic islets to demonstrate the cytoprotective effect of sericin on pancreatic islets (Morikawa et al. 2009; Ohnishi et al. 2012).

The process of islet transplantation consists of four steps: first, islets are isolated from the pancreas; second, the islets are purified, by rinsing and hand picking of the isolated islets; third, islets are preserved by culture preservation or cryopreservation; and fourth, islets are transplanted. In this study, we defined “isolation” as the process of isolating and purifying islets, as described in the first and second steps above. Morikawa et al. (2009) and Onishi et al. (2012) reported the effects of sericin in the preservation process; Morikawa et al. (2009) added sericin to culture medium, while Onishi et al. (2012) added it to frozen liquid. The objective of this study was to determine whether adding sericin to the isolation solution increased the quantity of pancreatic islets isolated. Our results suggest that the cytoprotection from sericin during isolation and purification could ensure that a sufficient number of islets are obtained from one donor for islet transplantation and make it possible to achieve a one donor—one recipient ratio.

## Materials and methods

### Experimental animals

Male Lewis rats (8–12 weeks old; Charles River Japan, Yokohama, Japan) were used in this study. They were weighed immediately before their use in this study. Diabetes was induced by intravenous injection of streptozotocin (60 mg/kg body weight; Sigma-Aldrich, St. Louis, MO, USA) through the caudal vein 7 days before transplantation. Rats showing non-fasting blood glucose levels of >350 mg/dL were used as transplant recipients. Blood glucose levels were determined using the glucose oxidase/peroxidase method on a Medisafe automatic analyzer (Terumo, Tokyo, Japan). The entire study was conducted in the animal facility of Fukui University in accordance with the regulation for Animal Research at Fukui University. The study protocol was reviewed and approved by the Animal Research Committee, Fukui University.

### Pancreatic islet isolation

Rats were anesthetized with isoflurane (Forane^®^; AbbVie Inc., North Chicago, IL, USA) and the abdomen was opened under aseptic conditions. The distal portion of the common bile duct was clamped and digestive enzymes were injected to distend the pancreas. For digestion of the pancreas, Hanks’ Balanced Salt Solution (HBSS; Sigma-Aldrich) which is the isolation solution was used by dissolving 1.8 mg/mL collagenase L (Nitta Zeratin, Osaka, Japan) and 0.3 mg/mL dispase II (Godo Shusei, Tokyo, Japan) followed by cooling. The pancreas was isolated and placed in a 50-mL centrifuge tube. After incubation in a water bath at 37 °C for 24 min, the tube was shaken well, and the pancreas was rinsed five times with isolating solution. Pancreatic islets were collected by hand picking.

For the sericin group, we added sericin to the isolation solution used in the isolation process.

### Pancreatic islet culture

The culture medium used in this study was prepared by adding 10 mM nicotinamide (Wako Pure Chemical Industries, Osaka, Japan) (Furuya et al. 2003), penicillin–streptomycin (Invitrogen™, Life Technologies Corporation, Carlsbad, CA, USA), and fetal bovine serum (final concentration: 10 %; Gibco^®^, Life Technologies Corporation) to RPMI-1640 medium (R8758; Sigma-Aldrich). For both the sericin and control groups, FBS without sericin was added to the culture medium. Pancreatic islets were transferred to a sterilized Petri dish and incubated at 37 °C for 2 days in a humidified atmosphere of 5 % CO_2_ and 95 % air.

### Determination of the volume at the time of pancreatic islet isolation

The collected pancreatic islets were placed in culture medium, and the number of pancreatic islets, longest diameter, and volume were determined for each sample at day 2 after starting the culture. The diameter of each islet was determined from the digital image (Digital Camera System: OLYMPUS DP70; OLYMPUS, Tokyo, Japan) under a stereoscopic microscope (Inverted System Microscope: OLYMPUS IX71; OLYMPUS) with 10 × magnification. The pancreatic islet volume was determined as islet equivalent (IEQ), and IEQ was calculated using the diameter of the islet (diameter of a 150 μm islet is defined as 1 IEQ) based on the assumption that the shape of the islet is spherical and every β-cell has the same functionality (Camillo et al. 1990; Fujita et al. 2011).

### Stereomicroscopic observation

Immediately after pancreatic islets were isolated, 30 islets per animal were placed in culture medium and examined under a stereoscopic microscope (Inverted System Microscope: OLYMPUS IX71; OLYMPUS).

### Light microscopic observation

Approximately 100 pancreatic islets were collected from each group to prepare 2-μM-thick sections for light microscopic observation.

### Insulin Secretion Assay

The amount of insulin secreted was determined at day 2 using a static incubation test. We used 12 rats in total with 6 animals in each group. Briefly, d-glucose was added to low-glucose medium (RPMI-1640 [R 1383: sugar free]; Sigma-Aldrich) to a final concentration of 3.3 mM, and pancreatic islets (diameter of 150 μm or more, 10 islets per group) of the control group and the 0.1 % sericin group were transferred to tubes containing 2 mL of the resulting solution and were pre-cultured at 37 °C for 1 h. Then, the pancreatic islets were incubated in low-glucose medium, high-glucose medium (final glucose concentration: 20 mM), and again in low-glucose medium for 1 h each (Korsgren et al. 1993). The insulin concentrations in the incubation media were determined by chemiluminescent automated method (CLIA) (Fabre et al. 2012).

### Islet transplantation

Diabetic recipient rats were used in the islet transplantation experiment. They were anesthetized with isoflurane. Approximately 800 pancreatic islets in the control group and the 0.1 % sericin group were cultured for 2 days and transplanted into the left renal subcapsular space of a diabetic recipient through a micropipette (200 μL) (Furuya et al. 2003). As all islets were derived from freshly isolated islets, islets transplanted into each group were equivalent. Non-fasting blood glucose levels in rats were monitored for 28 days and the transplant was removed by nephrectomy on the final day. Following nephrectomy, increases in non-fasting blood glucose levels were monitored for 7 days. In addition, islet grafts were used to prepare 2-μm-thick sections, which were stained for insulin using an immunohistochemical method (DAKO EnVision™ method), followed by hematoxylin & eosin staining (Sakura Finetek Japan Co., Ltd., Tokyo, Japan) for light microscopic observation.

### Labeling sericin with FITC and purification

Sericin (Seiren, Fukui, Japan) was labeled with Fluorescein isothiocyanate (FITC) (FITC Conjugation Kit; Sigma-Aldrich). After the addition of sericin to Dimethylformamide (DMF) (Sigma-Aldrich) and FITC, the solution was pipetted up and down until the FITC was completely dissolved and mixed well, then incubated at room temperature for 1 h. It was purified with PD Midi Trap G-10 (GE Healthcare UK Ltd., Chalfont St Giles, Buckinghamshire, England) and fractions were collected. The absorption spectrum of each fraction was measured with a Universal Plate Reader (Molecular Device SpectraMax M5, Sunnyvale, CA, USA). The fractions corresponding to FITC-labeled sericin were extracted and vacuum lyophilized (FZ-6PV, Labconco Corporation, Kansas City, MO, USA).

### Immunofluorescence staining

FITC-labeled sericin was diluted to a concentration of 0.1 % and added to the isolation solution used for pancreatic islet isolation. The obtained pancreatic islets were fixed in 4 % paraformaldehyde solution (paraformaldehyde; Wako) and excited at 480 nm using a confocal laser scanning microscope (TCS-SP2-AOBS; Leica).

Approximately 200 pancreatic islets that had been isolated in the same manner were collected to prepare 2-μm-thick sections (Murakami et al. 2000). One series of sections was stained for insulin (1:500; DAKO EnVision™ method) for light microscopic observation. Another series of sections was incubated with an Alexa 405 (1:100, goat polyclonal, anti-mouse IgG(H + L) secondary antibody; Life Technologies Corporation)-labeled anti E-cadherin antibody (1:100, mouse monoclonal, anti-E Cadherin IgG; abcam^®^, Cambridge, UK) and subjected to double immunofluorescence staining for FITC-labeled sericin and Alexa 405-labeled anti-E-cadherin. Alexa 405 and FITC were excited at 406 and 480 nm, respectively, and the sections were observed using a confocal laser scanning microscope.

### Scanning electron microscopic observation

One hundred pancreatic islets were collected from each group, dehydrated, incubated in isoamyl acetate, critical point dried, coated with gold, and observed under a scanning electron microscope (JSE-3690) at magnifications ranging from 100 × to 3000 × (Machida et al. 2013).

### Statistical analysis

The Mann–Whitney *U* test was used to compare the volume of pancreatic islets according to the body weight of rats, the insulin secretion levels and stimulation index in the insulin secretion assay, and non-fasting blood glucose levels of rats in the islet transplantation experiment. The *χ* ^2^ test was used for comparison of the number of pancreatic islets by the longest diameter. Differences were considered significant if the *p* value was less than 0.05.

### Animal assignment

In this study, the sericin and control groups refer to pancreatic islets that were isolated and purified using isolation solution with added sericin or no sericin, respectively. To determine the optimal concentration of sericin, the isolation solution was prepared containing 0.05, 0.1, or 0.2 % sericin. After isolation and purification, the body weight of rats, as well as the volume, number, and longest diameter of pancreatic islets were determined.

Insulin secretion level, islet transplantation, immunofluorescence staining pattern, and morphology were evaluated in two groups, i.e., the control group and the 0.1 % sericin group.

## Results

### Body weight of rats

There were no significant individual differences in body weight among the four groups, i.e., control, 0.05, 0.1, and 0.2 % sericin (control group: 299.5 ± 35.6 g, 0.05 % sericin group: 297.3 ± 34.5 g, 0.1 % sericin group: 296.8 ± 37.2 g, 0.2 % sericin group 297.7 ± 36.9 g).

### Volume of pancreatic islets

**Fig. 1:**
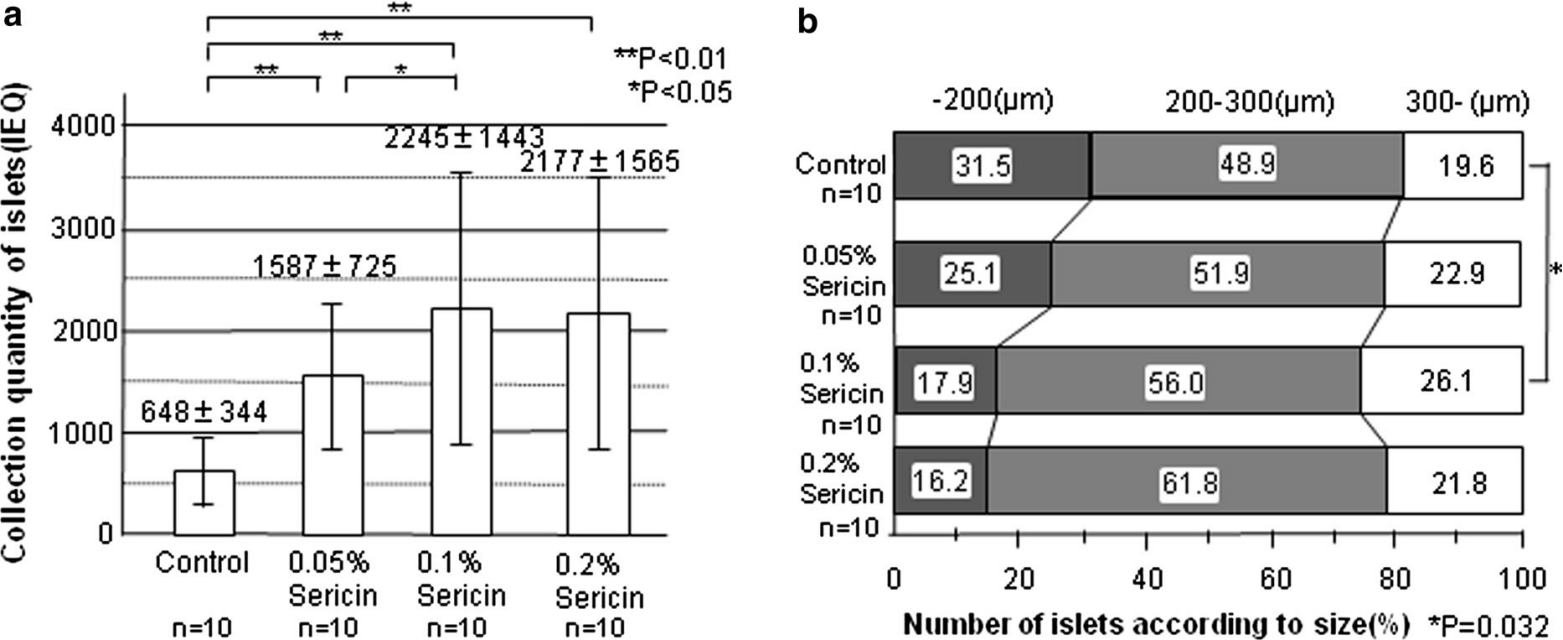
Volume and size of pancreatic islets. **a** Yield of pancreatic islets isolated according to the sericin concentration (n = 10). Values represent the mean ± SD. **b** Number of pancreatic islets isolated according to size were compared among the four groups (n = 10). The optimal sericin concentration was 0.1 %

The volume of pancreatic islets was significantly higher in the sericin groups than in the control group (Fig. 1a) (control group vs. 0.05 % sericin group: 648 IEQ vs. 1587 IEQ, *p* = 0.0057; vs. 0.1 % sericin group: 2245 IEQ, *p* = 0.00076; vs. 0.2 % sericin group: 2177 IEQ, *p* = 0.00058). Among the sericin groups, the largest volume was seen in the 0.1 % sericin group; it was significantly increased compared to the 0.05 % sericin group (0.05 % sericin group vs. 0.1 % sericin group: 1587 IEQ vs. 2245 IEQ; *p* = 0.039). These results indicated that the optimal concentration of sericin in the isolation solution is 0.1 %. For concentrations of sericin above 0.1 %, the volume of islets was not greater.

### Size of pancreatic islets

Pancreatic islets were divided into three groups by the longest diameter, i.e., ≤200, 200–300, and ≥300 μm, within each of the 4 groups. When the number of pancreatic islets in each group was correlated with the total number of pancreatic islets, the percentage of islets with a diameter ≥300 μm was significantly higher in the 0.1 % sericin group than the control group (control group vs. 0.1 % sericin group: 19.6 vs. 26.1 %; *p* = 0.032) (Fig. 1b). In addition, higher concentrations of sericin resulted in a lower percentage of islets with a diameter <200 μm. A higher concentration of sericin in the isolation solution tended to preserve the integrity of pancreatic islets so that they were not damaged.

### Morphology of isolated pancreatic islets

**Fig. 2:**
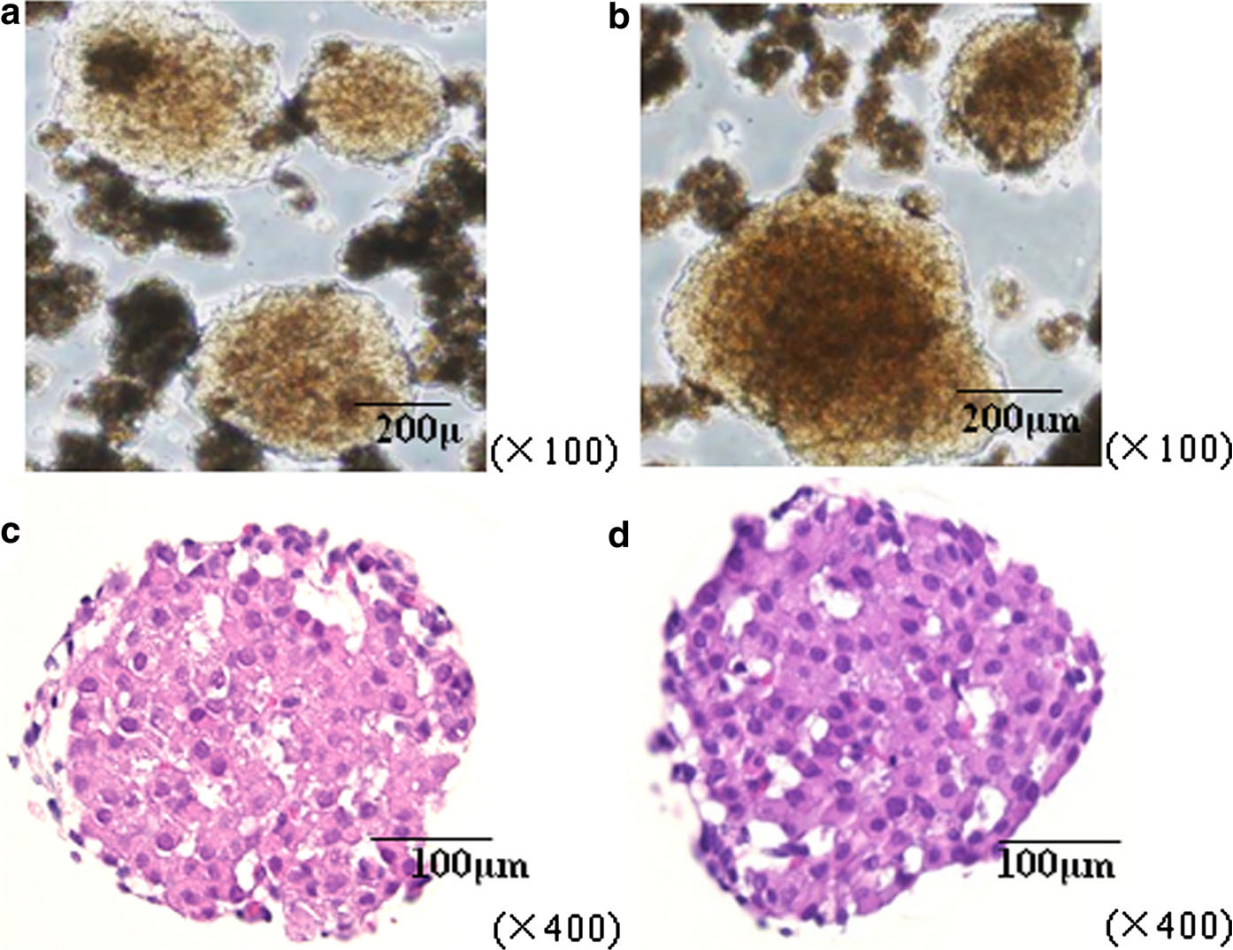
A micrograph of pancreatic islets immediately after isolation. Stereoscopic image ×100 **a** control group, **b** sericin group. Light microscopic image ×400 (hematoxylin & eosin staining), **c** control group, **d** sericin group

In both groups, isolated islets were brown and round or ellipsoidal, and pancreatic exocrine cells were scattered in the background as observed under a stereoscopic microscope. No significant differences in morphology were identified between islets isolated with or without sericin in the isolation solution (Fig. 2a, b). We sectioned islets in both groups and observed the cross section of islets under a light microscope. The islets displayed a well-preserved structure without significant differences between the sericin and control groups (Fig. 2c, d).

### Insulin secretion assay

Isolated islets from the control and sericin groups secreted an adequate amount of insulin during 20 mM (high) glucose stimulation (control group: 3.06 ng/mL/h; sericin group: 3.09 ng/mL/h). There were no significant differences in insulin secretion for the two groups (*p* = 0.47). The stimulation index indicates ability of the islets to regulate insulin; no significant difference was found between the groups (control group: 2.68; sericin group: 2.58; *p* = 0.98) (Table 1).

**Table 1:** The result of insulin secretion assay

Groups	Stimulant (glucose) (ng/mL/h per ten islets)			Stimulation index
	3.3 (mM)	20 (mM)	3.3 (mM)	
Control (n = 6)	1.44 ± 0.42	3.06 ± 0.91	1.20 ± 0.38	2.68 ± 0.84
Sericin (n = 6)	1.60 ± 0.72	3.08 ± 0.83	1.35 ± 0.52	2.58 ± 0.51
*p* value		*p* = 0.47		*p* = 0.98

Control group, pancreatic islets that were isolated using isolating solution without additive; sericin group, pancreatic islets that were isolated by adding 0.1 % sericin

Values represent the mean ± SD

### Islet transplantation

**Fig. 3:**
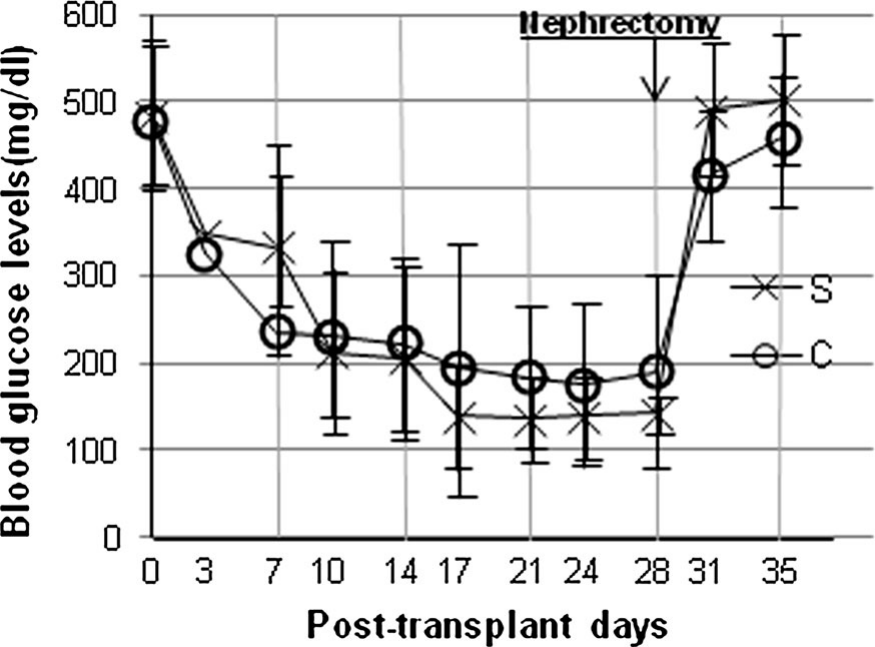
Islet transplantation. Non-fasting blood glucose levels in diabetic rats after transplantation of 800 islets in control group (c) (n = 6) or sericin group (s) (n = 6). There were no significant differences between the two groups

Blood glucose levels began to lower 7 to 10 days after islet transplantation, after which levels stabilized in both groups. All recipient rats reverted to the diabetic state after nephrectomy (Fig. 3). At 14–28 days, non-fasting blood glucose levels in transplanted rats in the sericin group tended to be lower than in the control group. However, there were no significant differences between the groups (*p* = 0.93). On the day 7, non-fasting blood glucose levels in the sericin group were higher than in the control group (Table 2). In other words, the insulin secretion in the islets from the sericin group appeared to normalize late, but showed no loss of function compared to islets isolated by conventional methods in islet transplantation in vivo. Of note, to obtain 800 islets, 3 donor rats were needed from the control group, but only 2 were required from the sericin group. This result suggests that the use of sericin could reduce the number of donors.

**Table 2:** Non-fasting blood glucose levels of diabetic rats after islet transplantation

Groups	Days after islet transplantation					
	0	7	14	21	28^a^	31
Control (n = 6)	476 ± 87.1	235 ± 106	222 ± 111	183 ± 82.6	189 ± 128	415 ± 80.0
Sericin (n = 6)	483 ± 80.6	333 ± 122	206 ± 104	137 ± 47.6	143 ± 22.4	491 ± 71.0
*p* value	*p* = 0.93	*p* = 0.18	*p* = 0.70	*p* = 0.094	*p* = 0.93	*p* = 0.094

Control group, pancreatic islets that were isolated using isolation solution without additive; sericin group, pancreatic islets that were isolated by adding 0.1 % sericin

Values represent the mean ± SD. Their units were ng/mL

The results at 3, 10, 17, 24 days were omitted

^a^At 28 days after islet transplantation, rats’ islet grafts were removed by nephrectomy

**Fig. 4:**
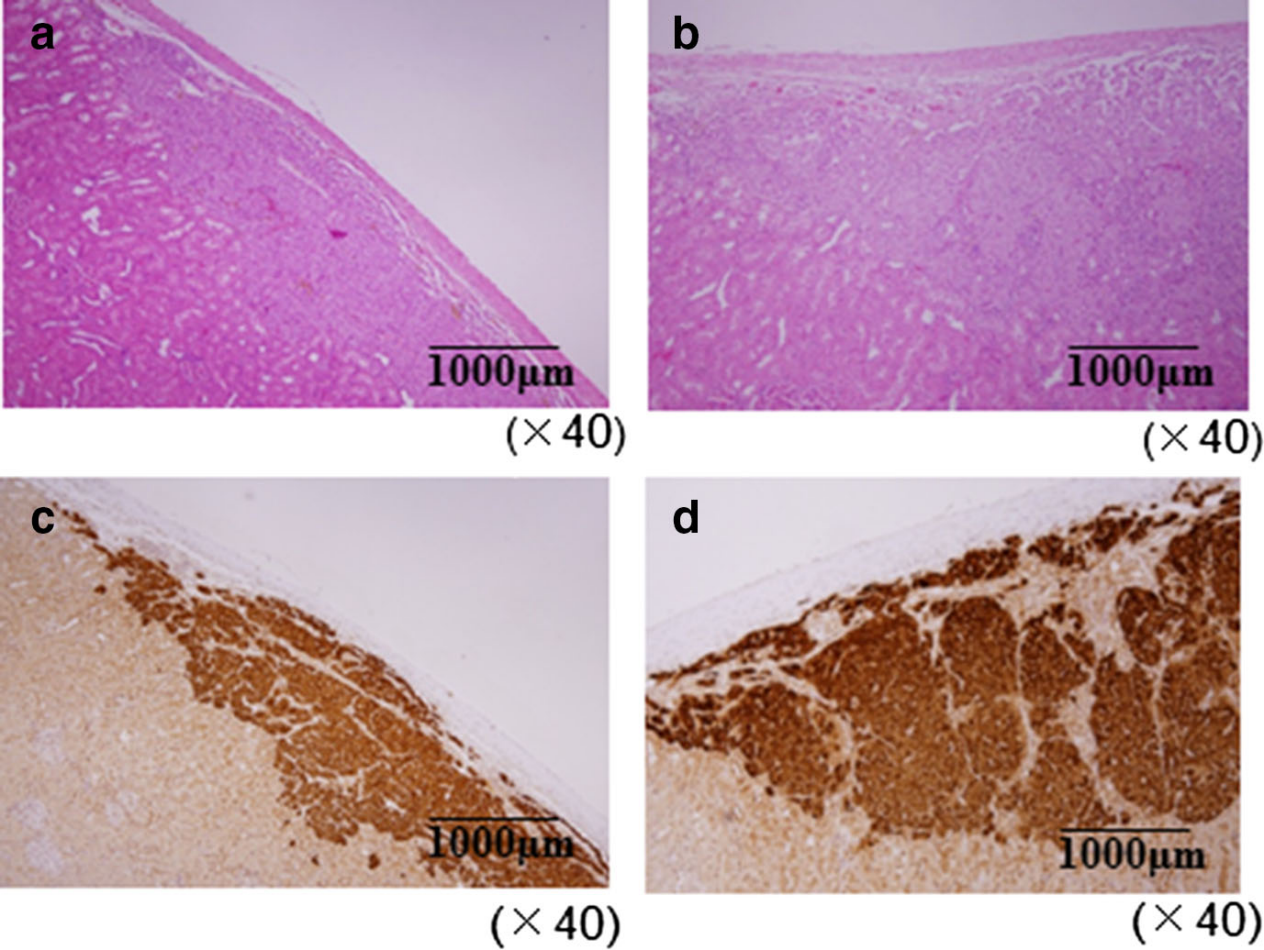
Islet grafts after nephrectomy. Islet grafts removed at 28 days after islet transplantation were examined with a light microscope. Histological image ×40 (hematoxylin & eosin stain), **a** control group, **b** sericin group. Immunohistochemical image ×40 (insulin stain), **c** control group, **d** sericin group

Islet grafts after resection at 28 days of transplantation showed a good structure under renal subcapsular space, and the tissue engrafted well and was stable despite its immunoreaction (Fig. 4a, b). Under light microscope, we observed numerous insulin-positive cells in islet grafts and no significant differences between the two groups. This showed that islet grafts secreted insulin in the control and sericin groups (Fig. 4c, d). We confirmed the good engraftment and numerous insulin-positive cells in islets in all cases for both groups.

### Distribution of sericin in pancreatic islets

**Fig. 5:**
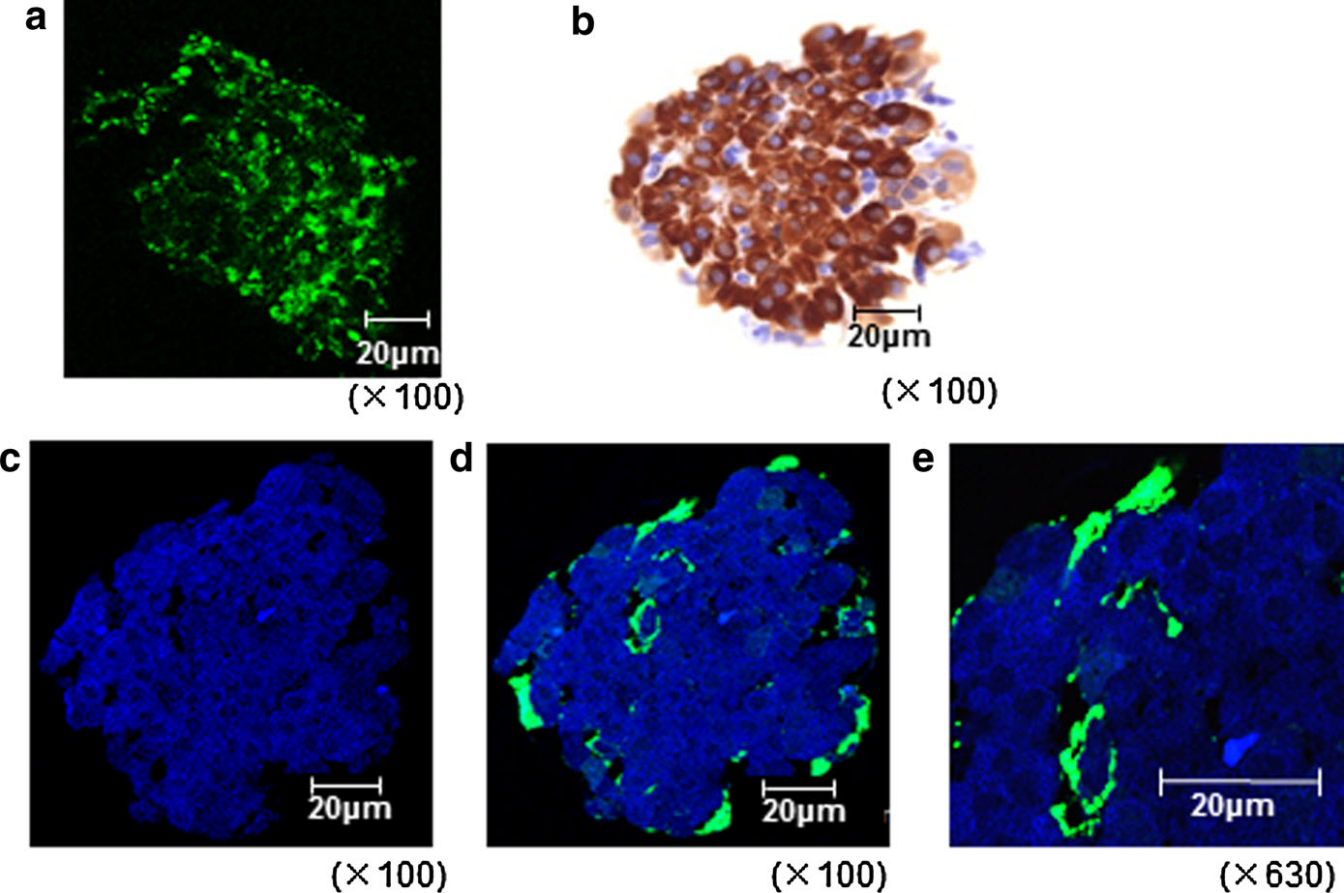
Immunofluorescence staining. Pancreatic islets that were isolated using fluorescein isothiocyanate (FITC)-labeled sericin. **a** Stereoscopic image ×100. Sectioned and stained pancreatic islets. Staining: **b** insulin ×100, **c** Alexa 405-labeled E-cadherin ×100, **d** Alexa 405-labeled E-cadherin and FITC-labeled sericin ×100 and **e** Alexa 405-labeled E-cadherin and FITC-labeled sericin ×630

Observation under a confocal laser-scanning microscope revealed that the degree to which FITC-labeled sericin adhered to pancreatic islets varied; however, it was only detected on the pancreatic islets (Fig. 5a). This indicated that the sericin had some influence on cytoprotection of pancreatic islets, but could not prove from this observation which the sericin attachment to the surface or entering into islet cells. E-cadherin is a membrane protein found in many cell types. In the sections of pancreatic islets, Alexa 405-labeled E-cadherin revealed the surface of the islet cells and outlined them (Fig. 5c). The distribution of FITC-labeled sericin coincided with the localization of E-cadherin on the surface of pancreatic islets and between the islet cells (Fig. 5d, e). This indicated that the sericin attached to the cell membrane of islet cells and was not taken up by cells. Images of pancreatic islets stained with an anti-insulin antibody are shown in Fig. 5b. This verified that the cells used in these experiments included β cells and were genuine islet cells.

### Electron microscopic observation

**Fig. 6:**
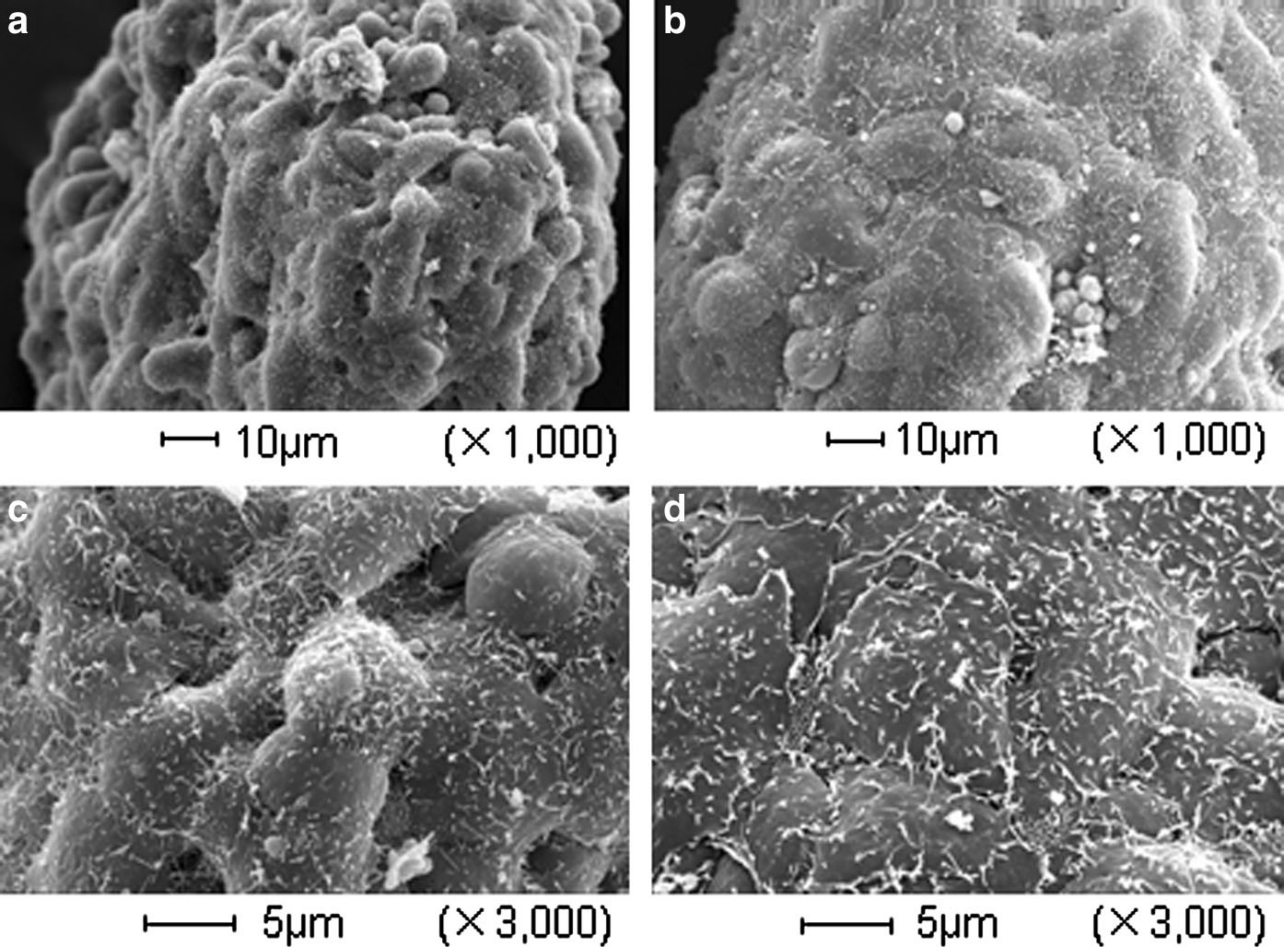
Electron micrograph of islets. **a** Control group, ×1000; **b** sericin group, ×1000; **c** control group, ×3000; **d** sericin group, ×3000

The morphology of pancreatic islets was evaluated using an electron microscope. The surface of the pancreatic islets of the control group was usually rough, whereas that of the pancreatic islets of the sericin group was smooth and the cells were flat (Fig. 6). Published electron micrographs show that the surface of intact islets is smooth, and the cells are flat when observed under an electron microscope (Ushiki 2013). The morphology of islets in the sericin group closely resembled that of the intact islets. This suggests that the morphology of islets in the sericin group was better maintained than in the control group.

These differences may not have been visible under the light microscope.

## Discussion

Sericin, a silk protein, is obtained from silkworms by purification of silk cocoons. There are numerous reports on the different properties of sericin. Recently, Terada et al. reported that sericin had a similar effect on the proliferation of mammalian cells, e.g., human epithelial cells (HeLa), human hepatoblastoma cells (HepG2), rabbit cornea keratocytes (RC4), and murine hybridoma cells, as bovine serum albumin (Sato et al. 2011; Terada et al. 2002). In addition, sericin possesses anti-oxidant activity by which it inhibits colon cancer and skin cancer (Sasaki et al. 2000; Zhaorigetu et al. 2003, 2007) and exerts anti-apoptotic effects (Takahashi et al. 2003). Due to these qualities, stock and cryopreservation solutions containing sericin for use in experiments have been marketed. Cosmetic products contain sericin because it possesses antioxidant activity and protects from ultraviolet damage (Zhaorigetu et al. 2003). Among the various properties of sericin, we have focused on its cytoprotective effects and examined its applicability to pancreatic islet transplantation.

Pancreatic islet transplantation is considered the most promising treatment strategy for Type 1 diabetes mellitus; however, this strategy has still some limitations and has not been widely used. Because cultivation or cryopreservation negatively affects the functionality of pancreatic islets, they are transplanted without subjecting them to these processes. In other countries, the time of death is adjusted in such a way that two or three donors are available at a time; however, because such regulation is not practiced in Japan, there is a shortage in donors and one donor-one recipient pancreatic islet transplantation should be used unless several brain-dead donors are available at the same time. If the technology of the culture and cryopreservation of pancreatic islets is well established, the isolated islets can be preserved for a long period of time which would allow for more time to be taken to carefully select recipients. Thus, the establishment of a method for the culture and cryopreservation of pancreatic islets would contribute to more effective islet transplantation. Furthermore, the ability to isolate more pancreatic islets from each donor increases the number of islets available for the transplant. In this study, we focused on increasing the efficiency of pancreatic islet isolation and studied whether cytoprotection by sericin can be applied to the isolation and purification processes. Damage caused by various factors, including ischemia, temperature change, osmotic pressure, chemical damages by collagenase and pancreatic enzymes, physical damages by shaking, and free radicals that occur during pancreatic islets isolation may reduce the volume of pancreatic islets (Maier 1986; Pileggi et al. 2006). To prevent the reduction in the volume of pancreatic islets caused by the above-mentioned damage-causing factors, an appropriate collagenase should be selected or pancreatic islets should be protected from the above-mentioned causes of damage during isolation. There are many reports on collagenases, but there are only a few reports demonstrating that addition of a protective substance during isolation increases protection of pancreatic islets and their yield. Tsukada et al. (2012) reported that ATP levels decreased in pancreatic tissue due to excessive collagenase digestion, but addition of serine protease inhibitors to the co-culture clearly showed protection of pancreatic islets. do Amaral et al. (2013) focused on reactive oxygen species that damage pancreatic islets and demonstrated that glutamine-ethyl-ester supplementation improved the viability of islets during the isolation process and during maintenance in culture before islet transplantation. Furthermore, Machida et al. (2013) reported that the use of a modified ET-Kyoto solution, i.e., ET-Kyoto/neutrophil elastase inhibitor (S-Kyoto), during islet isolation increased the yield and viability of pancreatic islets and that the volume of pancreatic islets was 2.5-time greater compared with the control group. In this study, the volume of pancreatic islets that were isolated was significantly increased by adding the silk protein sericin to the isolating solution. Based on the results of the insulin secretion assay and islet transplantation experiment, the endocrine function of islets in the sericin group was statistically comparable to that of the control group both in vitro and in vivo. In the islets transplantation, however, we noticed that the endocrine function of the sericin group could tend to be better than that of the control group. After islet transplantation, non-fasting blood glucose levels in the sericin group appeared to normalize late. This result was not negative but positive, because it suggested that islet cells damaged during transplantation released insulin in the initial period (0–14 days), which might lower glucose levels. In other words, it is possible that more cells were damaged during transplantation in the control group than in the sericin group. In addition, 7–14 days are required for revascularization of transplanted islets after transplantation (Nishimura et al. 2013), and glucose levels in the sericin group lowered late. This indicated that pancreatic islets isolated with the addition of sericin in the isolation solution could maintain stability and function during islet transplantation.

Cytoprotection by sericin has been demonstrated in various studies; however, the site of action of sericin has not been known. Islets isolated with FITC-labeled sericin in the isolation solution were observed to be sparsely covered with FITC-labeled sericin. The sericin adhered to the surface layer of islets, colocalizing with the cell membrane protein E-cadherin. In addition, in electron microscopic observation, the surface of islets from the sericin group was smooth, and the surface of dilapidated islets which were more observed in the control group than in the sericin group, was rough. Electron microscopic observation indicated that individual cells of damaged pancreatic islets protruded and the surface became rough. In contrast, the surface of intact islets is flat and smooth (Ushiki 2013). In the breakdown and destabilization of pancreatic islets, it is suggested that the islet cells could break free from the surface of the islets and be transformed from flat to spherical due to loss of cell–cell adhesion. This suggested that the cell–cell adhesion of smooth islet cells could be maintained. The islet morphology in the sericin group closely resembled that of an intact islet. This indicated that the cell–cell adhesion might be stronger, and the morphology could be better maintained than that of the control group. We propose that sericin might interact with E-cadherin and/or other membrane proteins directly, strengthen cell–cell adhesion, or transmit a signal into the cells inducing gene expression as the cytoprotective effect. The morphology of pancreatic islets is important because during islet transplantation, not only β cells but also α cells and δ cells are simultaneously transplanted. According to Nakai et al., when insulin-secreting cells produced by insulin gene transfer into 3T3-L1 cells were transplanted into mice with streptozotocin-induced type 1 diabetes, mice died due to hypoglycemia, indicating that a regulatory system to control blood glucose levels is required (Nakai et al. 2008). It is considered that the system may be regulated by pancreatic islets, indicating that pancreatic islet transplantation requires a critical amount of pancreatic islets with normal morphology that is preserved during isolation.

Protection of pancreatic islets by sericin is characterized by the maintenance of their morphology. In this study, the morphology of pancreatic islets was maintained during isolation by adding sericin to the isolation solution, and Morikawa et al. (2009) and Ohnishi et al. (2012) reported the maintenance of islets during culture and cryopreservation by the addition of sericin to culture medium and frozen liquid. The advantages of the addition of sericin during the isolation process and the use of sericin in cultures and cryopreservation in the preservation process could be to maintain the morphology of pancreatic islets and to increase the volume of transplantable pancreatic islets isolated from one donor. In addition, the method is simple and can be easily applied. Similar to the culture and cryopreservation experiments, our results from the insulin secretion assay and the islet transplantation experiment show that there was no significant difference in the islets’ endocrine function with addition of sericin. Silk fibers used in surgery also contain small amounts of sericin and no adverse effects were reported when it is incorporated into the body. However, in the case of pancreatic islet transplantation, a large volume of pancreatic islets is transplanted into the portal vein. Therefore, future studies should investigate whether sericin induces liver damage or infection. In the future, transplantation of porcine pancreatic islets, which are closer to those of humans, will be performed to determine whether similar effects can be obtained.

We propose that sericin could contribute to islet therapy by enhancing islet stability.

## Conclusion

This study indicated that sericin did not affect the digestion process, protected pancreatic islets from cytoarchitectural damage during isolation and purification, did not affect islet endocrine function, and increased the volume of the yield of pancreatic islets.
